# Molecular and immunohistochemical characterization of intestinal macrophages subsets in goldfish

**DOI:** 10.1038/s41598-026-48801-y

**Published:** 2026-05-06

**Authors:** Giacomo Zaccone, Doaa Mokhtar, Marco Albano, Alessio Alesci, Gioele Capillo, Partha Sarathi Tripathy, Marialuisa Aragona, Maria Cristina Guerrera, Jose Manuel Icardo, Hailah M. Almohaimeed, Sebastian Marino, Eugenia Rita Lauriano, Jorge Manuel de Oliveira Fernandes

**Affiliations:** 1 Department of Veterinary Sciences, University of Messina, 98168 Messina, Italy; 2 Department of Cell and Tissues, Faculty of Veterinary Medicine, Assiut University, Assiut, 71526 Egypt; 3 Department of Histology and Anatomy, School of Veterinary Medicine, Badr University in Assiut, Assiut, Egypt; 4 Department of Chemical, Biological, Pharmaceutical and Environmental Sciences, University of Messina, 98166 Messina, Italy; 5 Sea in Health and Life Srl, c/o Department of Chemical, Biological, Pharmaceutical and Environmental Sciences, University of Messina, Capo Peloro, Messina, Italy; 6 College of Fisheries, Rani Lakshmi Bai Central Agricultural University, Uttar Pradesh, 284003 Jhansi, India; 7 Zebrafish Neuromorphology Lab, Department of Veterinary Sciences, University of Messina, 98168 Messina, Italy; 8 Department of Anatomy and Cell Biology, Poligono de Cazona, Faculty of Medicine, University of Cantabria, 39011 Santander, Spain; 9 Department of Basic Science, College of Medicine, Princess Nourah Bint Abdulrahman University, P.O.Box 84428, 11671 Riyadh, Saudi Arabia; 10 Department of Renewable Marine Resources, Institut de Ciencies del Mar (ICM-CSIC), 08003 Barcelona, Spain; 11 Faculty of Biosciences and Aquaculture, Nord University, 8049 Bodø, Norway

**Keywords:** Intestinal immunity, CSF, CD14, CD86, BMP2, Goldfish, Immunology, Neuroscience

## Abstract

Macrophage-colony stimulating factor (M-CSF) plays a crucial role in the proliferation and differentiation of the monocyte-macrophage lineage across vertebrates. In this study, we identified and characterized two CSF1 paralogues and their corresponding receptor genes in the intestine of the goldfish (*Carassius auratus*), showing sequence homology with known teleost species. Immunohistochemical analysis confirmed the presence of CSF, its receptor CSF-R1, and bone morphogenetic protein 2 (BMP2) in intestinal macrophages. These macrophages were localized within mucosal, submucosal, and muscularis layers, suggesting distinct functional subtypes. Quantitative PCR analysis revealed differential gene expression patterns, with *csf1a*, *csf1b*, and *csf1ra* highly expressed in the brain, while *csf1rb* transcripts were predominant in the intestine. Immunophenotypic characterization using CD14 and CD86 markers further demonstrated macrophage heterogeneity. Additionally, BMP2-expressing macrophages were observed in the muscularis externa, implying a potential role in neuromuscular regulation. These findings provide novel insights into the molecular and immunohistochemical profiles of goldfish intestinal macrophages, highlighting their potential role in immune responses and gut homeostasis.

**Supplementary Information:**

The online version contains supplementary material available at 10.1038/s41598-026-48801-y.

## Introduction

The intestine is the primary organ responsible for nutrient absorption and represents the largest mucosal surface in vertebrates, which is essential for the induction of mucosal and systemic responses and the maintenance of metabolic-immune homeostasis^1^. Many studies pointed to the enteric nervous system (ENS) as a crucial player in maintaining the immune homeostasis of the gastrointestinal microenvironment^2,3^. Crosstalk between ENS and the immune cells, mainly macrophages, T cells, and innate lymphoid cells (ILCs) revealed a key regulation in intestinal tissue programming, homeostasis, function, and inflammation^3–5^.

Macrophages carry out diverse functions in the mammalian intestine that vary according to their anatomical location^6^. For example, macrophages that localize to the tissue situated directly underneath the gut epithelium, known as lamina propria, contribute to immune defense against pathogenic bacteria^6^. Recently, Zaccone et al.^7^ observed the expression of acetylcholine, its nicotinic receptor alpha 7 (alpha-7-NAChR), and the antimicrobial peptide piscidin-1 (Pis 1) by macrophages in gut epithelium. These data demonstrate that the ENS controls intestinal immune responses via direct stimulation of the resident macrophages^8^. In the mammalian gut, a distinct group of macrophages localizes between the circular and longitudinal muscle layers in the tissue region known as muscularis externa (MMs), and express genes that are distinct from lamina propria^6^. They regulate the activity of smooth muscle cells^9^ and secrete soluble factors, such as BMP2, which interact with enteric neurons that control smooth muscle cell activity^3^.

A colocalization of macrophages and enteric neurons is reported in the submucosal and myenteric plexuses in the fish gut using double immunolabeling cell markers^7^. Enteric neurons contribute to the maintenance of muscularis macrophages. Correspondingly, macrophages directly affect neuronal homeostasis via the release of the BMP2, which binds to its receptor BMPRII on enteric neurons^3^. The bidirectional interplay between MMs and enteric neurons is essential to maintain proper tissue function under physiological conditions^8^. Macrophages are highly heterogeneous tissue-resident immune cells that perform a variety of tissue-supportive functions conforming to their microenvironment, including the efficient response towards tissue damage and microbial insults^10^ as demonstrated in the fish intestine^11^. Consequently, tissue-resident macrophages are extremely heterogenous in terms of origin and function and possess a unique transcriptome that allows them to fulfill niche-specific functions^12^.

CSFs are a family of cytokines that regulate the production, differentiation, and function of various blood cell lineages, including macrophages. In the mammalian intestine, two main types of CSFs, namely macrophage colony-stimulating factor (M-CSF) and granulocyte–macrophage colony-stimulating factor (GM-CSF), are involved in the regulation of intestinal macrophages^13^. M-CSF, also known as CSF-1, is a key regulator of macrophage development and survival. It is produced by various cell types, including stromal cells, fibroblasts, vascular smooth cells, and endothelial cells in the intestine^14,15^. M-CSF binds to its receptor, CSF-1R, which is expressed on the surface of macrophages. This binding activates downstream signaling pathways, including the PI3K–Akt pathway, which promotes macrophage survival and proliferation, the MAPK/ERK pathway, which supports differentiation and proliferation, and the JAK/STAT pathway, which regulates gene expression involved in macrophage activation and immune responses, ultimately leading to the proliferation, survival, and differentiation of intestinal macrophages^13,16–19^. GM-CSF, on the other hand, is produced by immune cells such as activated T lymphocytes, macrophages, dendritic cells, and natural killer (NK) cells. In addition, innate lymphoid cells (ILCs) are a major source of intestinal GM-CSF^20,21^. It acts as a potent stimulator of macrophage function and is involved in the regulation of immune responses. GM-CSF binds to its receptor, GM-CSFR, which is expressed on the surface of macrophages. This binding triggers signaling cascades that enhance the phagocytic activity, antigen presentation, and cytokine production of intestine macrophages^21^.

CSFs and CSFRs were characterized in some teleost fish including cyprinids, zebrafish and goldfish^22^. However, we have a limited understanding of the molecular mechanisms and signaling pathways involved in CSF.

CSF and Colony-stimulating factor receptor (CSFR) function by which the macrophages regulate intestinal neuromuscular activity and gut motility in all vertebrate classes. In the case of teleosts, there is an additional layer of complexity due to the presence of multiple *csf* paralogues and their receptors, which arose from the teleost-specific whole genome duplication event that is estimated to have occurred during the early Permian period^23^.

Despite the recognized importance of intestinal macrophages in vertebrate immunity, our understanding of their molecular identity, spatial distribution, and functional heterogeneity in teleosts remains limited. In particular, the roles of colony-stimulating factors (CSFs) and their receptors (CSFRs) in sustaining resident macrophage populations and coordinating neuro-immune interactions in the fish gut have not been comprehensively investigated. Furthermore, the potential involvement of BMP2in intestinal macrophage function is virtually unexplored in teleosts.

Goldfish (*Carassius auratus*) offer a valuable model for such investigations due to their evolutionary position, availability, and previously reported immune–neuroendocrine features^7,24^. This study integrates molecular, phylogenetic, and immunohistochemical approaches to characterize CSF/CSFR and BMP2 expression in distinct intestinal macrophage subsets of goldfish. By doing so, we provide the first detailed description of these markers in teleost intestinal macrophages, revealing novel insights into their possible roles in immune regulation, gut homeostasis, and neuroimmune crosstalk. These findings contribute to bridging the knowledge gap between mammalian and teleost intestinal immunity and may inform strategies for improving fish health in aquaculture.

## Materials and methods

### Ethics statement

Handling and care of animals followed the ethical principles indicated by the European Union Directive (63/2010/EU) on the use of animals for scientific purposes, following the ARRIVE guidelines. This study was approved by the ethics committee at the University of Messina.

### Animals and sample collection

Ten mature goldfish (*Carassius auratus*) of approximately 9–12 months old, each measuring 7.0 cm in total length were obtained by a local supplier. The fish were housed in aerated aquaria in the laboratory at 25°C with an LD 12/12 photoperiod for four weeks. Fish were fed ad libitum twice a day with commercial aquarium fish food (Goldfish flakes, Tetra, Germany). Prior to sampling, the fish were euthanized by immersion in MS-222 (3% tricaine) for 15 min. The brain and posterior intestine were carefully dissected, snap-frozen in liquid nitrogen and stored at -80 °C for molecular analysis. The remainder alimentary tract was dissected and prepared for histological and immunohistochemical analysis, as detailed below.

### Quantification of gene expression by qPCR

The identification of *csf* and *csfr* paralogues in the goldfish genome was done through homology-based searches. Specifically, known sequences of *csf* and *csfr* from other species of order Cypriniformes were used as queries to perform NCBI-BLAST searches against the goldfish genome. The sequences of colony-stimulating factors 1a (*csf1a*), colony-stimulating factor 1b (*csf1b*), colony stimulating-factor 1 receptor a (*csf1ra*) and colony stimulating-factor 1 receptor b (*csf1rb*) were successfully identified, confirming their presence as paralogues in the goldfish genome. These paralogues were characterized by their high sequence similarity and conservation with previously known counterparts from other species from order Cypriniformes.

Five brain and five intestinal samples were used as biological replicates for the qPCR of *csf1a*, *csf1b*, colony stimulating-factor 1 receptor a (*csf1ra*), and colony stimulating-factor 1 receptor b (*csf1rb*). For every qPCR reaction, two technical replicates were employed. The primers were designed for these genes using NCBI’s Primer-BLAST tool. NetPrimer (Premier Biosoft, Palo Alto, USA) was then used to assess these primers for secondary structures such as hairpins, repetitions, and both self and cross-dimers (Table 1). The QuantiTect reverse transcription kit (Qiagen, Hilden, Germany) was used to reverse transcribe 1 µg of total RNA from each sample in accordance with the manufacturer’s instructions. This cDNA was then utilized as the template for qPCR after being diluted 20 times with nuclease-free water. SYBR green on the LightCycler® 96 Real-Time PCR System (Roche Holding AG, Basel, Switzerland) was used to perform the qPCR reactions. The thermocycling conditions were as follows: an initial denaturation at 95°C for 10 min, followed by 35 cycles at 95°C for 20 s, 60°C for 30 s, and 72°C for 10 s. The relative expression of the chosen genes was used to evaluate the PCR efficiency^25^. Normalization factors were derived from the geometric mean of two reference genes (*actb* and *ef1a*) and relative expression quantities factored in the geometric mean of both reference genes and the PCR efficiencies^26^. The qPCR analytical method used factored in the geometric mean of both reference genes and the PCR efficiencies. A two-sample t-test was used to determine the statistical difference (P < 0.05) following the confirmation of the normality and homogeneity of variance assumptions.

**Table 1:** List of qPCR primers used in the present study along with their respective Ensembl IDs.

Gene	Forward primer	Reverse primer	Product length (bp)	Ensembl gene ID
*csf1a*	GGCCCATGTAAGCACTCTGT	CAGTATGTGAGGGACCGCTG	159	ENSCARG00000025558
*csf1b*	GGACCACCTGCTTCAACTGA	AACAGTTCAAGCACCCTCGG	141	ENSCART00000101371
*csf1ra*	TGTTTGGCCATCGAGAGCAT	CGGGGAGAGTCGAATGTACG	150	ENSCART00000058559
*csf1rb*	TTCCTGGTGTGTTGAGCTCC	TGTGCACACTGACCACTCTC	131	ENSCART00000041926
*actb*	CCTTCCTTCCTGGGTATGG	TCCTTCTGCATACGGTCAG	158	ENSCART00000054067
*ef1a*	CAGGTCATCATCCTGAACCAC	AACGACGGTCGATCTTCTC	121	ENSCART00000064806

### Phylogenetic tree reconstruction and visualization

To investigate the evolutionary relationships of *csf1a*, *csf1b*, *csf1ra* and *csf1rb* in goldfish (*Carassius auratus*) and other related teleost species, nucleotide sequences were retrieved from NCBI GenBank database (https://www.ncbi.nlm.nih.gov/genbank/) and aligned using MEGA11 software^27^. Phylogenetic trees were constructed using the Neighbor-Joining (NJ) method with 1,000 bootstrap replicates to assess nodal support. The aligned sequences were exported in FASTA format, and the resulting tree topology was saved in Newick (.nwk) format.

For the visualization of the phylogenetic tree alongside the multiple sequence alignment, the R packages ggtree^28^. treeio, Biostrings, and msa were used. The Newick tree file was imported using read.tree from the ape package, and the corresponding aligned sequences were read using readDNAStringSet from Biostrings. To ensure consistency, tip labels of the tree were cross-validated with the sequence names using intersect. The ggtree function was used to plot the phylogenetic tree, and sequence alignments were added alongside using msaplot with an offset of 11.8 units to avoid overlap between the tree and alignment blocks. All the analysis and visualizations were performed in R v4.4.2.

### Tissue preparation and confocal immunofluorescence

Intestinal tissues were fixed in 4% paraformaldehyde in phosphate-buffered saline (PBS; pH 7.4) for 6–8 h. Following fixation, samples were rinsed in PBS, dehydrated through graded ethanol series, cleared in xylene, and embedded in paraffin (Paraplast, McCormick Scientific, St. Louis, MO, USA). Serial sections were cut at a thickness of 5–10 µm using a rotary microtome (Leica RM2135, Germany) and mounted on gelatin-coated slides.

Sections were first deparaffinized in xylene and rehydrated through descending grades of ethanol to PBS. After rehydration, non-specific binding sites were blocked by incubating the sections with 2.5% bovine serum albumin (BSA) in PBS for 1 h at room temperature. The sections were then incubated overnight at 4°C in a humidified chamber with primary antibodies diluted in PBS containing 1–2% BSA. Each section was processed for double immunolabeling using appropriate combinations of primary antibodies. After washing in PBS, sections were incubated with secondary antibodies (Alexa Fluor 488 donkey anti-mouse IgG and Alexa Fluor 594 donkey anti-rabbit IgG; Molecular Probes, Invitrogen, USA) for 1 h at room temperature in the dark. Following additional PBS washes, sections were mounted using Vectashield mounting medium (Vector Laboratories, Burlingame, CA, USA) to prevent photobleaching and coverslipped.

Negative controls were performed by omitting the primary antibodies. Rat intestinal tissue and goldfish gill sections were used as heterologous and homologous positive controls, respectively, to validate antibody reactivity. Details of all antibodies, including source, host species, and working dilutions, are provided in Table 2**.**

**Table 2:** Antibodies used in this study:

Antibody	Supplier	Dilution	Animal source	Cat. Num
M-CSF	Abcam Limited, Cambridge, UK	1:200	Rabbit	ab52864
CSF-1-R	Abcam Limited, Cambridge, UK	1:200	Rabbit	ab183316
Alexa Fluor 488 donkey anti-mouse IgG FITC conjugated	Molecular Probes, Invitrogen, Eugene, U.S.A	1:300	Donkey	A-21202
Alexa Fluor 594 donkey anti-rabbit IgG TRITC conjugated	Molecular Probes, Invitrogen, Eugene, U.S.A	1:300	Donkey	A-21207
CD14	Abcam Limited, Cambridge, UK	1:200	Mouse	ab181470
CD86	Abcam Limited, Cambridge, UK	1:200	Rabbit	ab53004
Α-Tubulin	Santa Cruz Biotechnology, Inc., Dallas, TX, USA	1:200	Mouse	sc-8035
Calbindin	Novocastra Laboratories Ltd, Newcastle, UK	1:200	Mouse	NCL-CALBINDIN
BMP-2	Santa Cruz Biotechnology, Inc., Dallas, TX, USA	1:200	Rabbit	sc-9003

All primary antibodies were selected based on reported cross-reactivity in teleost studies and their use in previous morphological analyses of fish immune and intestinal tissues^29^. In this study, specificity was further supported by epitope conservation analysis ( Table S1), appropriate tissue distribution patterns, and the absence of staining in negative controls. The application of antibodies to identify the fish’s autonomic innervation, neuroepithelial cell systems, and immune cell systems was the main focus of these investigations.

### Image acquisition and processing

Sections were examined and images were captured using a Zeiss LSM DUO confocal laser scanning microscope (Carl Zeiss MicroImaging GmbH, Jena, Germany, Europe) equipped with a META module. This microscope has two argon lasers (458 and 488) and two helium–neon lasers (543 and 633 l). Each image was digitized into a 2048 × 2048-pixel array with an 8-bit resolution. Optical slices of fluorescence samples were made using argon (458 nm) and helium–neon (543 nm) lasers with scanning speeds of 1 min and 2s. Zen 2011 (LSM 700 Zeiss software, Oberkochen, Germany, Europe) was used to improve the photos. In order to avoid deterioration, every picture was taken as quickly as possible. To create the figure composite, digital photos were cropped using Adobe Photoshop CC (Adobe Systems, San Jose, CA, USA).

### Quantitative analysis of macrophage subsets

Confocal z-stacks (step size 0.5–1.0 µm) from goldfish intestinal sections double-immunolabeled for CD14 and either CSF1, CSF1R, or BMP2 were analyzed to determine the proportion of CD14 + cells exhibiting immunoreactivity for these markers. CD14-immunoreactive cells were manually counted in at least five non-overlapping regions of interest (ROIs) per section across a minimum of 10 sections from three individual fish. Counts were performed across mucosal, submucosal, and muscular layers, and data from all layers were pooled for each fish to calculate an overall percentage per marker. Cells were considered double-positive when overlapping fluorescence signals were confirmed within the same optical plane in merged z-stack images. Percentages were calculated as the number of double-immunoreactive cells relative to the total number of CD14-immunoreactive cells per ROI. Data are presented as mean percentages ± standard error, with individual fish values plotted to illustrate biological variability.

## Results

### Phylogeny and transcript levels of csf1a, csf1b, and csf1ra and csf1rb

**Fig. 1:**
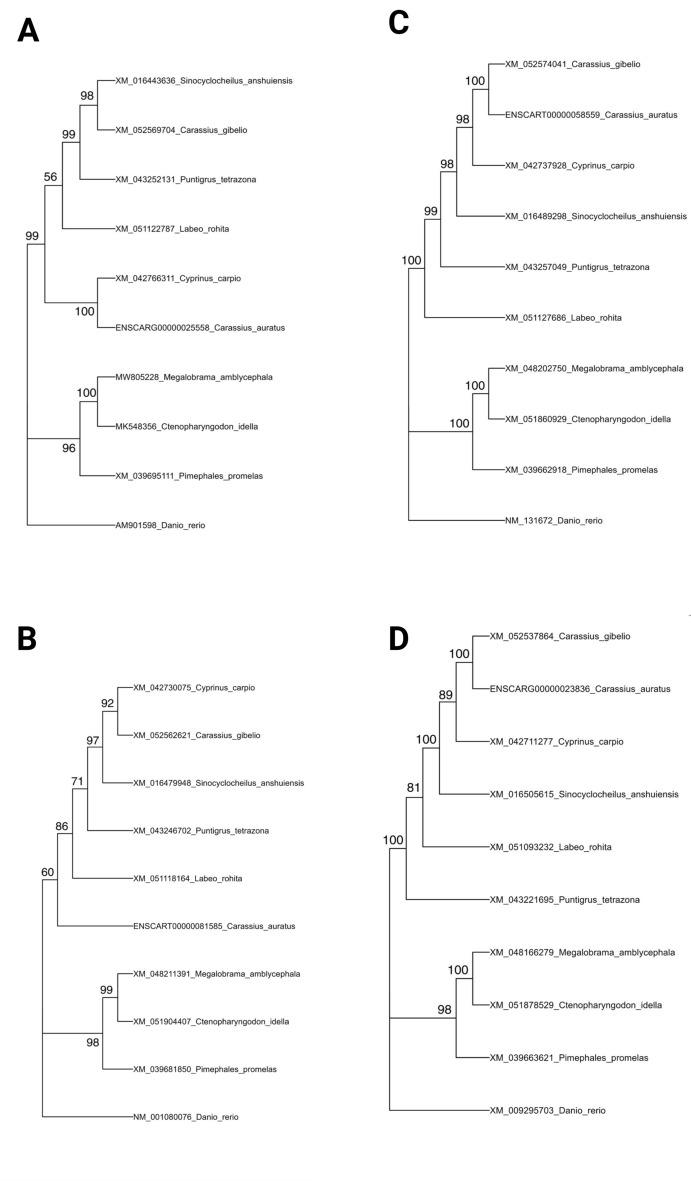
Phylogenetic relationship and sequence alignment of (**A**) *csf1a,* (**B**) *csf1b*, (**C**) *csf1ra,* and (**D**). *csf1b* genes in goldfish (*Carassius auratus*) and related cyprinid species. The phylogenetic tree was constructed using the Neighbor-Joining (NJ) method. Bootstrap values (based on 1,000 replicates) are shown at the nodes.

We have identified and characterized two *csf1* paralogues (*csf1a* and *csf1b*) and their corresponding receptor genes (*csf1ar* and *csf1br*) in the intestine of *C. auratus*. Both csf paralogues had a high degree of conservation amongst cyprinid taxa ( Fig. S1). Their phylogenetic reconstruction revealed that *C. auratus csf1a* clustered with *Cyprinus carpio*, instead of with its closest relative, *C. gibelio*. Similarly, *C. auratus csf1b* was more closely related to *Labeo rohita* instead of *C. gibelio*. The *csf* receptors *csf1ar* and *csf1br* were also highly conserved amongst cyprinid taxa and the *C. auratus* genes were grouped with the *C. gibelio* orthologues, as expected (Fig. 1). The amino acid aligned sequences corresponding to each taxon are displayed alongside the tree, with color-coded amino acids in Fig. S1.

**Fig. 2:**
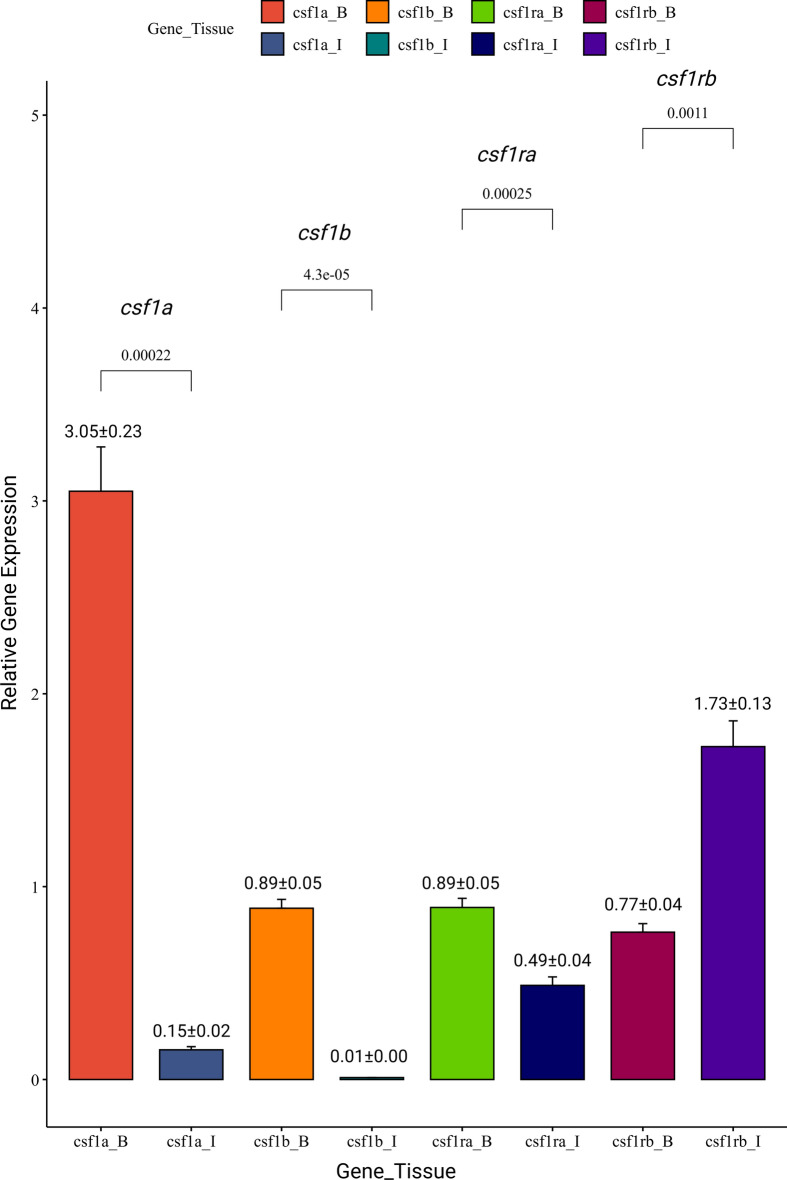
Relative gene expression of *csf1a, csf1b, csf1ra,* and *csf1rb* in goldfish brain and intestine tissues. Bar plots represent gene expression levels in the brain (B) and intestine (I). Statistical significance was determined using a t-test, with p-values indicated above the respective bars in square brackets. Data are presented as mean ± SE.

The relative expression of *csf1a*, *csf1b,* and *csf1ra* was significantly (p < 0.05) higher in the brain as compared to the intestine (Fig. 2). However, the relative expression of *csf1rb* was found to be expressed at lower levels in the brain as compared to the intestine (Fig. 2). Among all the four genes examined, the *csf1a* transcripts were found at higher levels in brain tissue (Fig. 2), whereas *csf1rb* expression was predominant in intestine tissue compared to all the four genes analyzed in the present study (Fig. 2).

### Immunohistochemistry (Double immunolabeling methods)

#### Expression of CSF-1 and CSFR: CSF1-CD14 and CSFR1-CD14

**Fig. 3:**
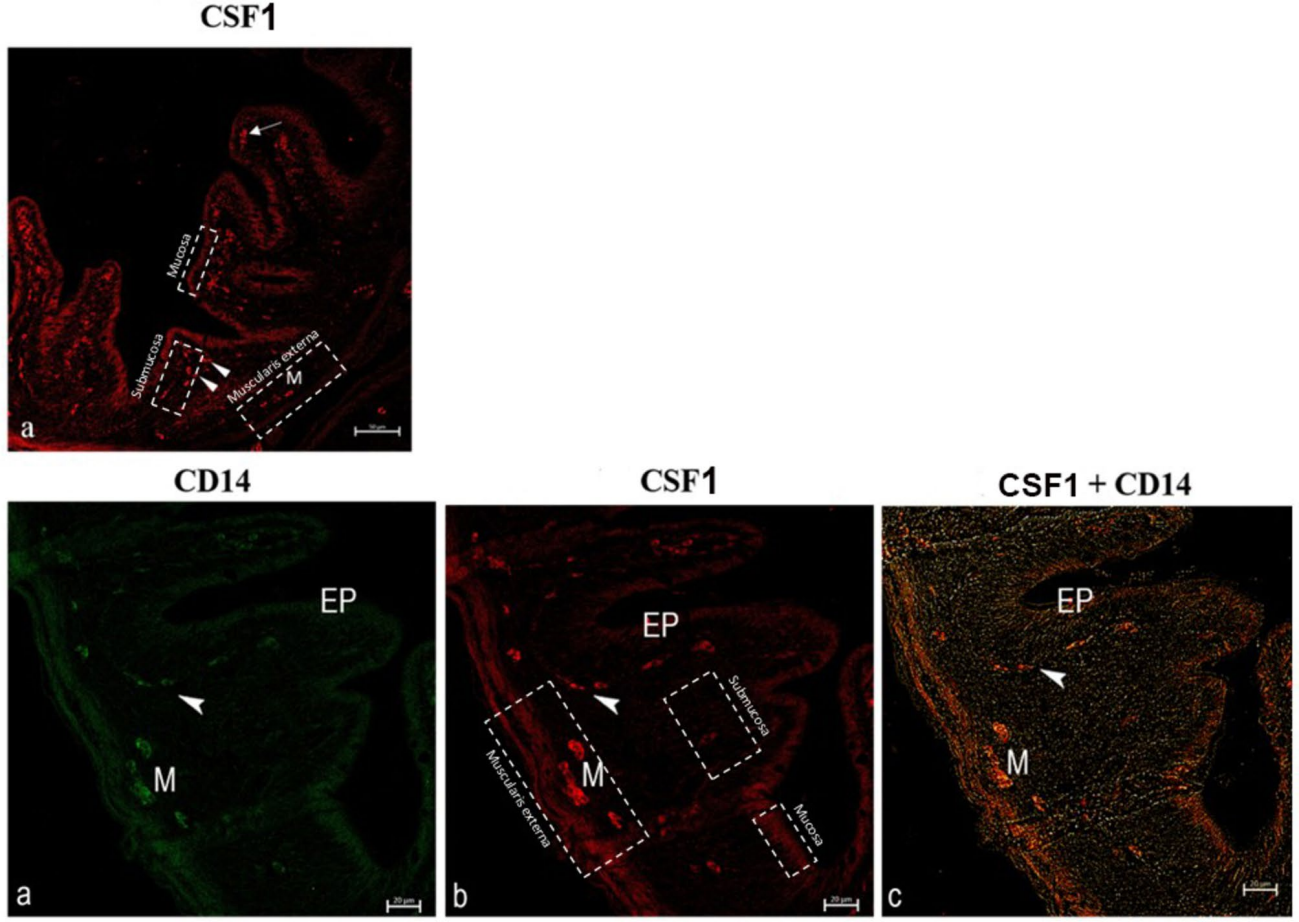
Confocal images of goldfish middle intestine. Double immunolabeling with antibodies against CSF1 and CD14. (**a**) Confocal image highlighting the CSF1 + cells in the lamina propria (arrows), submucosal layers (arrowheads) and muscularis (M). (**b**–**d**). Macrophages in the lamina propria (arrowheads), the mucosal epithelium (EP), muscularis (M) contain immunoreactivity for CD14 and CSF1. CSF1 is shown in red fluorescence, while CD14 is shown in green fluorescence.

**Fig. 4:**
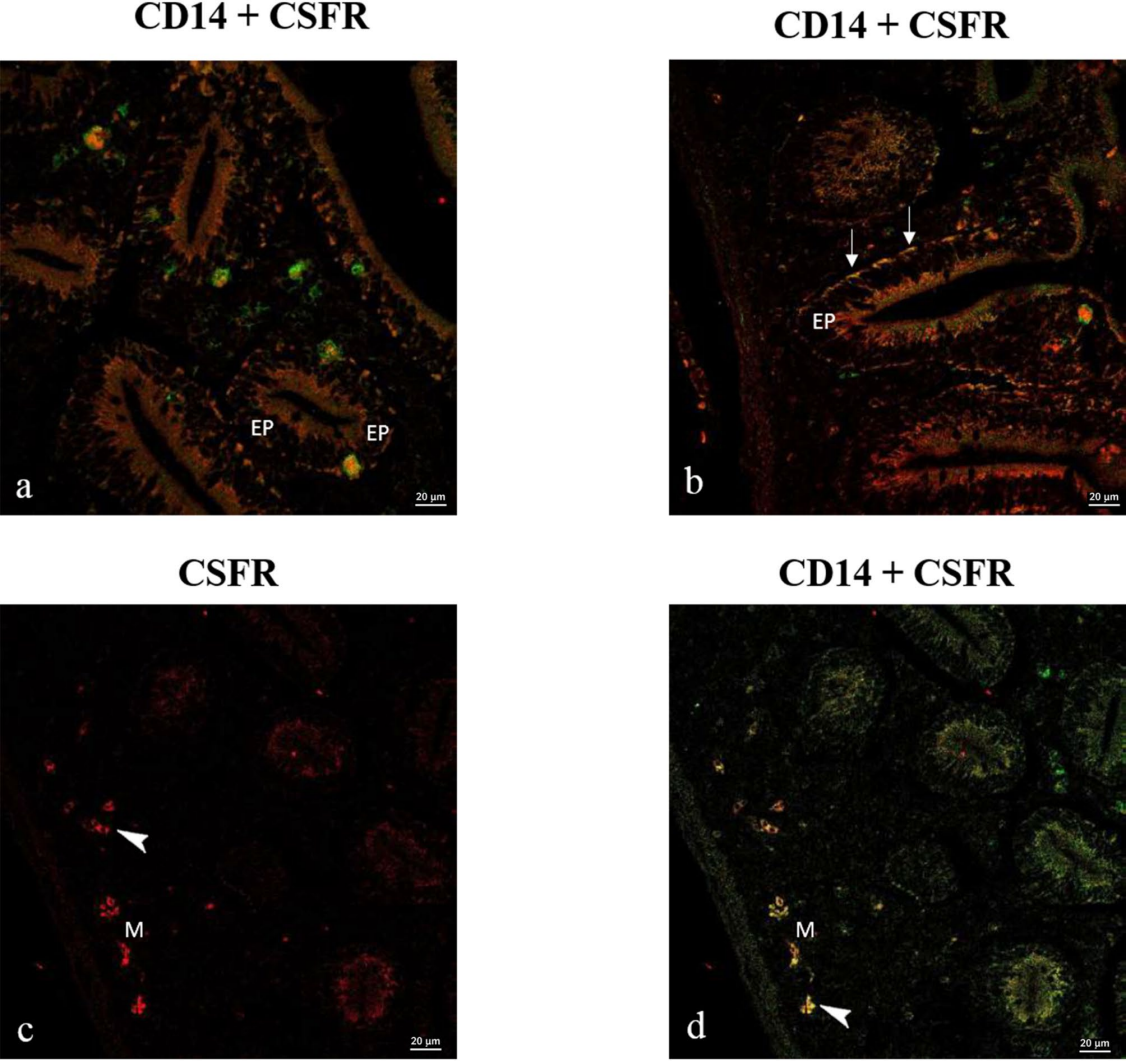
Double immunolabeling with antibodies against CSFR and CD14. (**a**) Merged image depicting the presence of macrophages in the mucosal epithelium (EP) and submucosal layer. (**b**) Merged image showing the presence of macrophages in the mucosal epithelium (EP) and their alignment in the lamina propria (arrows). (**c**, **d**) Red and merge channels showing aggregated macrophages (arrowheads) in smooth muscle layers (M). CSFR is shown in red fluorescence, while CD14 is shown in green fluorescence.

The goldfish intestine is a complex layered structure that includes a mucosa, submucosa and a muscularis externa (Fig. 3). The intestinal mucosa is populated by three subsets of macrophages. These subtypes were classified by the expression of multifunctional growth factors and their localization in mucosal, submucosal, and muscularis externa layers (Fig. 3). We found that these cell subtypes are found in the epithelial layers, lamina propria, subepithelial layers and packed in muscle layers (Figs. 3, 4). CSFR immunoreactivity partially overlapped with CD14-like macrophages, while additional CSFR-positive cells were observed within epithelial layers. (Fig. 4).

#### Expression of BMP2: BMP2-CD14

**Fig. 5:**
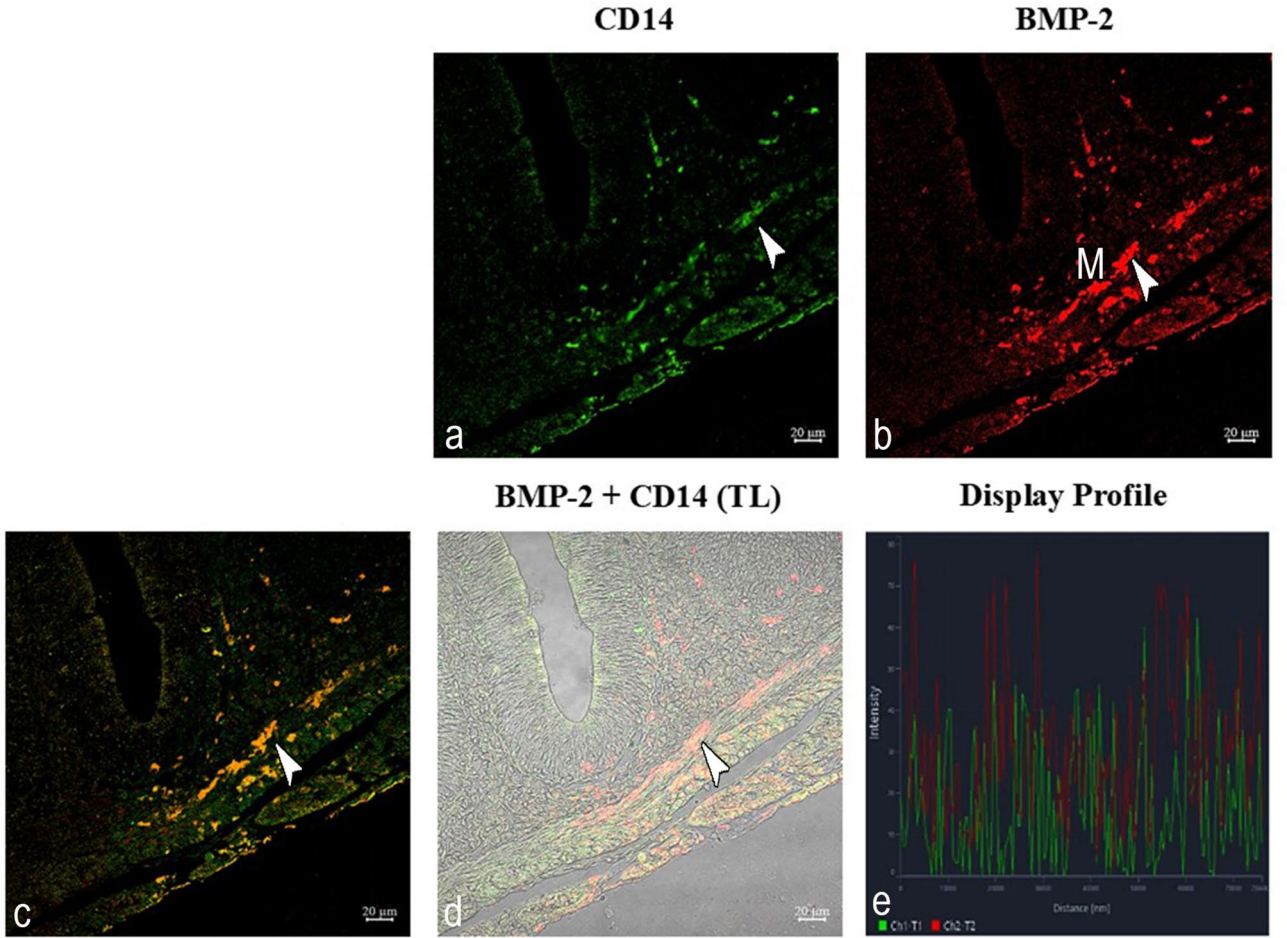
Double immunolabeling with antibodies against BMP2-CD14. (**a**, **b**) A distinct subpopulation of macrophages (arrowheads) containing BMP2 immunoreactivity is found in the circular muscle (M) of the goldfish intestine (red channel). BMP2 is not expressed with CD14 (green channel). (**c**, **d**) MCs (arrowheads) are immunoreactive to BMP2 and CD14. TL: Transmitted light. (e) The colocalization of antibodies is confirmed by the ‘display profile’ function.

Double immunolabeling with antibodies against CD14 and BMP2 enabled the observation of two distinct populations of macrophages in the intestinal mucosa, lamina propria, submucosal layers, and muscle layers. CD14-immunoreactive cells and BMP2 immunoreactivtiy mainly localized in the epithelial and submucosal layers, those immunoreacting to BMP2 resided in the muscle layers (Fig. 5a, b). The outer epithelial cell layers also contained BMP2 immunoreactivity (Fig. 5b). Double-label overlay images (CD14 + BMP2) emphasized co-localization, particularly in clusters within the tunica muscularis (Fig. 5c, d). The “display profile” feature verifies the antibodies’ colocalization (Fig. 5e).

#### Expression of calbindin: calbindin-CD86

**Fig. 6:**
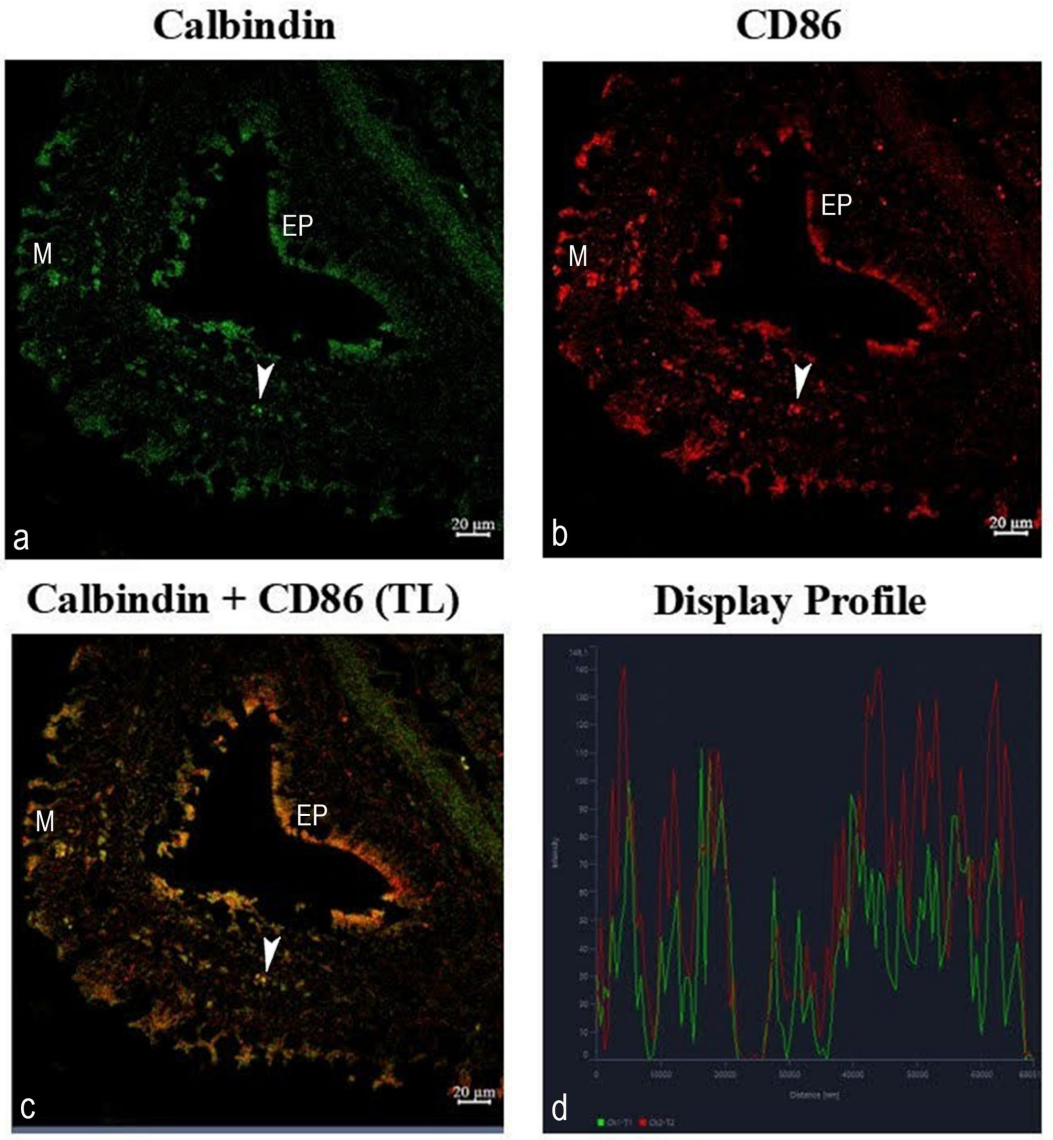
Confocal images of goldfish middle intestine. Double immunolabeling with antibodies against calbindin and CD86. (**a**) Demonstration of a fluorescence pattern of the two markers in cell subsets of epithelial (EP) and subepithelial macrophages (arrowhead). Note the presence of macrophages in muscles (M). (**b**) A distinct cell subset is labelled in red by CD86 in the intermediate to cell surface layers of the intestinal epithelium (EP). (**c**) A macrophage phenotype population is also seen in the smooth muscle layer (M) co-expressing the two markers (merged image). (**d**) The colocalization of antibodies is confirmed by the ‘display profile’ function. Green and red lines respectively indicate fluorescent signal curves of calbindin & CD86.

CD86- and calbindin-immunoreactive cells were detected within the lamina propria, submucosa, and muscular layers. The distribution appeared regionally variable rather than uniformly dense (Fig. 6a, b). A variable epithelial-associated immunoreactive signal was observed; however, the cellular identity of these CD86 + cells cannot be definitively determined (Fig. 6b). Two phenotypes were found. Macrophages double labelled with an antibody to calbindin and CD86 were tightly packed and seen with the layers of smooth muscle and submucosal layers (Fig. 6c). Cells positive for the antibody to CD86 were found in the surface epithelial layers near the mucous cells (Fig. 6b), or exposed to the epithelial cell surface.

#### Macrophages contact swollen varicosities on axons: Tubulin-CD86

**Fig. 7:**
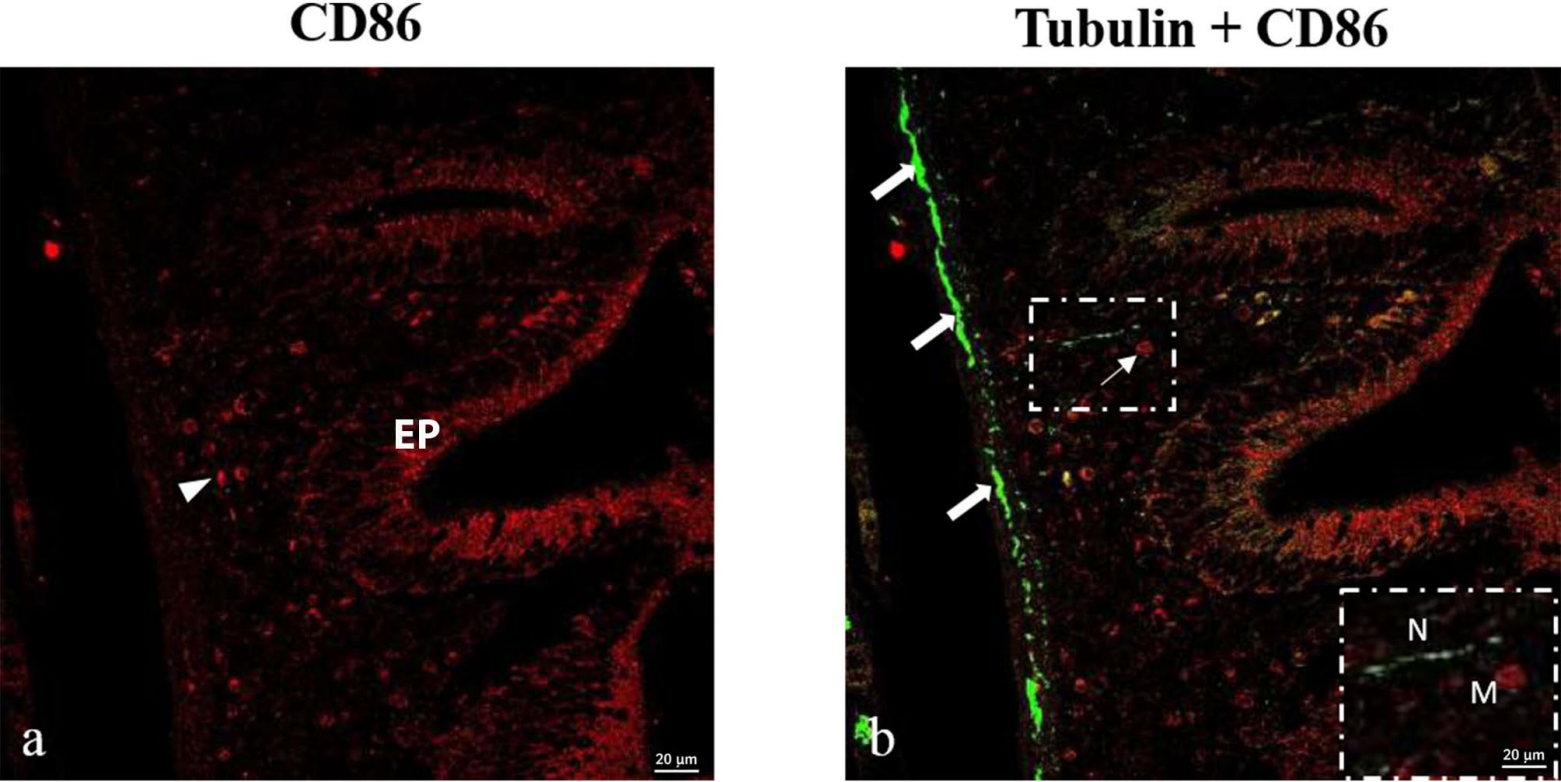
Confocal images of goldfish middle intestine. Double immunolabeling with antibodies against tubulin and CD86. (**a**) CD86 + cells in the middle to outer cell layers of the intestinal epithelium (EP). (**b**) A subset of macrophages (thin arrows) makes contact with tubulin-positive axons (thick arrows) that course through the submucous plexus and circular muscle. The inset represents a digital enlargement of the boxed region in the main panel and is provided only to facilitate visualization of the area of interest; it does not correspond to an independently acquired higher-magnification image. N indicates nerves or axons, and M indicates a macrophage in the vicinity of nerves. CD86 is shown in red fluorescence, while tubulin is shown in green fluorescence.

CD86-immunoreactive cells were observed within the muscular layer in proximity to tubulin-positive axons; however, consistent direct cellular contact was not evident (Fig. 7a, b). The cells were observed in contact with both swollen varicosities or normal-sized varicosities. These axons might be the sites of no synaptic neurotransmitter release. However, we have not tested in the present study these axons for tyrosine hydroxylase. Tubulin antibodies label axons within the layer of muscularis as the myenteric plexus.

#### Colocalization analysis of CSF1R and CD86 immunoreactivity

**Fig. 8:**
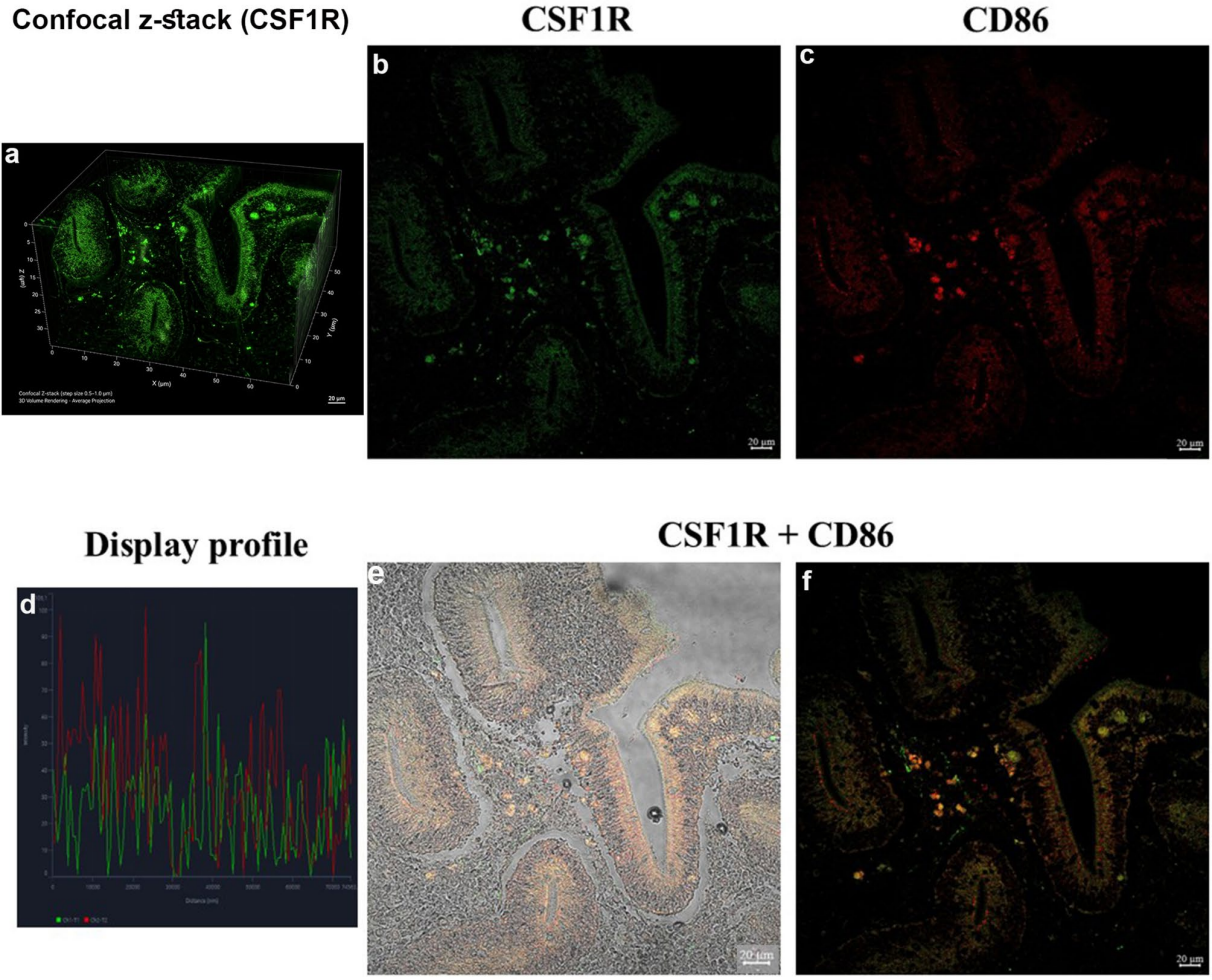
Colocalization analysis of CSF1R and CD86 immunoreactivity: (**a**) Three-dimensional confocal reconstruction of goldfish intestinal tissue. The image represents a volume-rendered projection generated from a confocal z-stack (step size 0.5–1.0 µm), showing the spatial architecture of intestinal folds and the distribution of fluorescently CSF1R-labeled cells. Axes (X, Y, Z) are indicated in micrometers. (**b**–**f**) Colocalization analysis of CSF1R (green) and CD86 (red) signals demonstrates partial overlap in selected cells within the lamina propria and submucosal compartments. Merged images indicate that while a subset of cells co-express both markers (yellow signal), other cells display single-marker immunoreactivity, reflecting cellular heterogeneity.

Three-dimensional reconstruction of confocal z-stack images revealed the spatial organization of intestinal structures and associated immunoreactive cells. Numerous fluorescently CSF1R-labeled cells were distributed throughout the epithelial lining and underlying lamina propria, with a higher density observed along the mucosal folds (Fig. 8a). Colocalization analysis of CSF1R and CD86 immunoreactivity revealed partial overlap within distinct cell populations in the intestinal mucosa and submucosa (Fig. 8 b-f). Confocal z-stack evaluation and merged channel analysis demonstrated that a subset of CSF1R⁺ cells also exhibited CD86 signal, whereas other CSF1R⁺ or CD86⁺ cells appeared singly labeled. The degree of overlap varied across intestinal layers and was more evident in the lamina propria compared to epithelial regions.

#### Quantitative analysis of confocal images

**Fig. 9:**
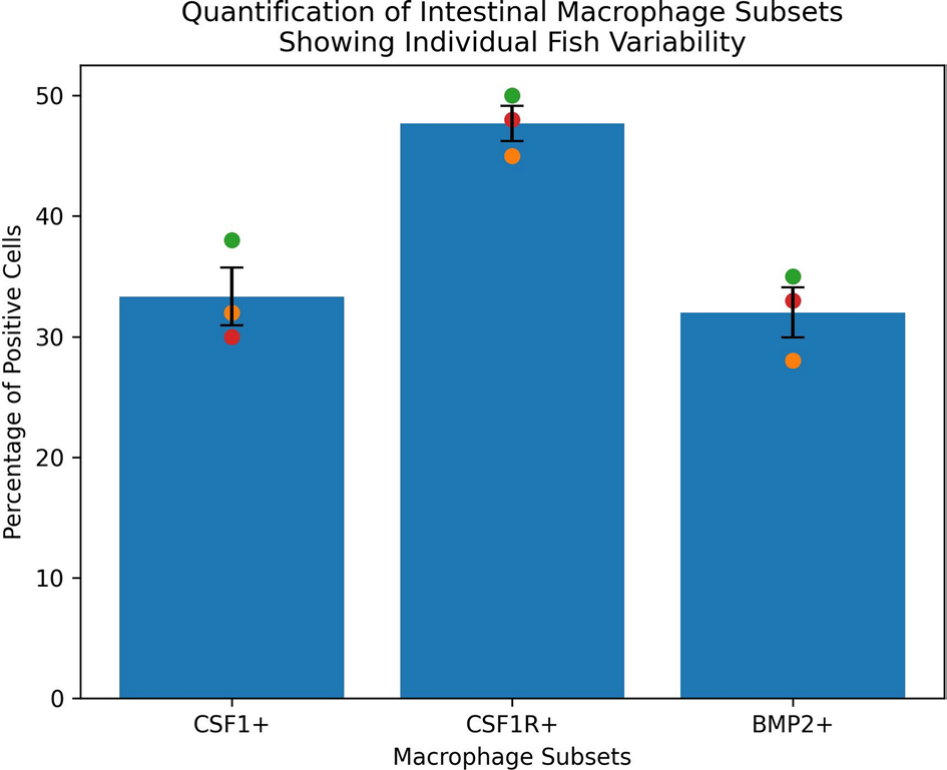
Quantification of intestinal immune cell subsets showing individual fish variability. Bar graph illustrating the percentage of CD14-like cells exhibiting immunoreactivity for CSF1, CSF1R, or BMP2 in goldfish intestine. Bars represent mean percentages calculated from three individual fish, with error bars indicating standard error. Colored dots represent individual fish values. CSF1R-immunoreactive cells constituted the largest proportion of the analyzed population, followed by CSF1 and BMP2. The data demonstrate inter-individual variability while maintaining a consistent distribution pattern among markers.

Quantitative analysis of intestinal immune cell subsets revealed measurable proportions of CD14-like cells exhibiting immunoreactivity for CSF1, CSF1R, and BMP2 (Fig. 9). The highest proportion was observed for CSF1R-immunoreactive cells, representing approximately 47–48% of the analyzed CD14-like population. CSF1-immunoreactive cells accounted for approximately 33%, while BMP2-immunoreactive cells represented approximately 32% of the total counted cells. Individual fish variability was evident across all markers, although the relative distribution pattern remained consistent, with CSF1R⁺ cells predominating over CSF1⁺ and BMP2⁺ subsets. Error bars indicate inter-individual variation among sampled fish. These data demonstrate heterogeneity within the intestinal immune cell population and suggest differential representation of cells exhibiting immunoreactivity to the examined markers.

#### Validation of antibody specificity

Epitope alignment demonstrated strong conservation between the supplier-reported immunogen sequences and predicted goldfish orthologs for CSF1R (91% identity) and BMP2 (85% identity) (Supplementary Table S1). CD14 showed lower conservation (~ 60%), and we therefore refer to its immunoreactivity as “CD14-like.”

Negative controls (no primary antibody) produced no staining, confirming that the immunofluorescent signals were specific to primary antibody binding ( Fig. S2). The distribution of CSF1⁺, CSF1R⁺, and BMP2⁺ cells was consistent with known macrophage niches in teleost fish. Colocalization analysis revealed partial overlap between CSF1R and CD86 signals, supporting macrophage identity (Fig. 8). Quantitative assessment across sections (Fig. 9) confirmed consistent labeling proportions, strengthening confidence in specificity despite the absence of goldfish-specific pre-absorption controls. Autofluorescence was excluded based on the absence of signal in no-primary controls, the discrete cellular localization of fluorescence, and the preservation of signal patterns across optical z-sections.

## Discussion

Based on their localization, intestinal resident macrophages are classified into three major groups: the mucosal macrophages lying in mid and outermost cell epithelial layers, the highly phagocytic lamina propria macrophages, which represent the first line of defense against pathogens and act primarily as innate immune effector cells, and the muscularis macrophages, which represent the dominant immune cell population in the muscular layer and closely and bi-directionally interact with the ENS and also with smooth muscle cells^30^.

Tissue-resident intestinal macrophages are specialized phagocytes in fish^11^ that carry out supportive functions during tissue development, homeostasis, and regeneration. In mammals, tissue resident macrophages are extremely heterogeneous in terms of origin and function and possess a unique transcriptome that allows them to fulfill niche-specific functions^12,31,32^. Vertebrate macrophages possess a rich repertoire of potent pre-formed antimicrobial molecules stored within their granules and lysosomes^33^. Fish intestinal macrophages are a source of the antimicrobial peptide, piscidin 1 and nitric oxide^7,34^ that contribute to critical effector mechanisms in limiting the growth of fish pathogens.

Limited studies have been performed using antibodies to teleosts CSF and CSF-1R^24,35,36^. Recently the expression and functional properties of the goldfish CSF-2 gene were reported^22^. Both molecular analyses and immunohistochemical antibodies against CSF-1 and its type III tyrosine kinase receptor, CSF-1R resulted as specific marker for goldfish macrophages^24^. Since macrophages are found across all metazoan, it is not surprising that these molecules that regulate macrophage development are also conserved.

CSF-1R sequences have been identified in a number of teleost species including puffer fish^37,38^, rainbow trout^39^, gilthead seabream^40^, zebrafish^41^, and goldfish^42^. Also as emphasized by Hanington, et al.^42^, goldfish appear to independently regulate their development in a manner unique from the mammalian system. (1) they are capable of producing their endogenous growth factor and progenitor cells can develop into fully functional monocytes and macrophages in vitro and (2) senescence phase macrophages produce a soluble form of the CSF-1R, sCSF-1R, through alternative splicing that is believed to be involved in the regulation of CSF-1 signaling through mCSF-1R^41^. While recombinant goldfish CSF-1 has been shown to induce monocyte proliferation and differentiation^42^ and aid in the long-term survival of mature macrophages in culture^24^. Our gene expression results show a distinctive tissue pattern. Specifically, *csf1rb* was downregulated in the brain but upregulated in the intestine, consistent with previous observations in zebrafish and gilthead seabream where *csf1r* paralogues display tissue-specific expression reflecting niche specialization of macrophage populations^35,41^. This enrichment in intestinal tissue likely supports a high-turnover, barrier-associated macrophage population adapted to constant microbial exposure. Studies in mammalian systems have demonstrated that macrophages reside in the brain as microglia and that primitive macrophages developed during embryogenesis giving rise to the microglia in adults^43^. However, while it seems likely that CSF-1R would be involved in goldfish microglia development, further studies are required^44^. CSF1 immunoreactivity was detected in proximity to CD14 + cells, suggesting possible local interactions; however, additional cellular sources of CSF1 within the intestinal tissue cannot be excluded.

It should be noted that we did not determine the sex of the sampled goldfish, as they were obtained outside the breeding season and external dimorphism is minimal in this period. Therefore, our dataset cannot assess sex-dependent differences in CSF/CSFR or BMP2 expression. Studies in zebrafish and tilapia have shown that immune gene expression may differ between sexes^45^. Future investigations should incorporate sex identification to examine whether such dimorphism exists in goldfish intestinal immune gene expression.

The observed upregulation of CSF1R in the intestine suggests a potential role in local macrophage survival or functional adaptation within the intestinal microenvironment; however, direct evidence for a self-maintaining macrophage population was not examined in the present study. Muscularis macrophage-like cells were consistently observed in close association with neural processes, suggesting a tissue-resident pattern; however, the potential regulatory influence of the enteric nervous system on their phenotype in the goldfish intestine has not yet been investigated. CSF and CSFR are important factors for the differentiation of monocytes into macrophages and their subsequent activation in higher vertebrates^16,17,46^. In terms of gene expression (for instance CSFR1b gene), profiles of lamina propria include genes responsible for initiating an immune response than muscularis externa where genes involved in tissue structure support were prominent^47^.

The presence of both CSF1 and CSF1R immunoreactivity within the intestinal tissue raises the possibility of local paracrine signaling; however, co-expression at the single-cell level was not examined in this study^48,49^. Unfortunately, there is no evidence in literature dealing with macrophage CSF regulating immune response against pathogenic infection in teleost fish. Our data demonstrate that a subset of CD14-like macrophages exhibits CSF1 immunoreactivity, suggesting that intestinal macrophages may contribute to local CSF1 production. However, we do not exclude additional cellular sources within the intestinal epithelium or stromal compartments, consistent with mammalian systems where CSF1 is primarily produced by non-hematopoietic cells. The objectives of future studies are also to examine how the signaling through the CSF-1 and CSF-1R mediates the proliferation, differentiation, and activation of macrophages and their progenitors in the goldfish intestine. This study is, to our knowledge, the first to describe BMP2-expressing macrophages in the intestine of any teleost fish. The presence of BMP2 in muscularis externa macrophages suggests a role in neuromuscular regulation, drawing parallels with mammalian gut macrophage–neuron interactions. This finding opens a new avenue for exploring how BMP2 might influence macrophage polarization and tissue repair in fish.

## Limitations and future directions

A key limitation of the present study is the reliance on commercially available antibodies developed against mammalian antigens. Although sequence conservation analysis of the reported immunogen regions supports potential cross-reactivity with goldfish proteins, definitive biochemical validation (e.g., Western blotting against recombinant goldfish proteins, peptide blocking assays, immunoprecipitation with mass spectrometry, or transfected goldfish cell systems) was not performed. Therefore, while the observed staining patterns are anatomically and cellularly consistent with macrophage-associated localization, the results should be interpreted as immunoreactivity consistent with the targeted proteins rather than absolute confirmation of antigen identity. The commercial anti-CSF1 antibody used in this study has not been validated in genetic knockout models. Therefore, although sequence conservation and anatomical distribution support potential cross-reactivity, definitive biochemical validation remain necessary. Future work will include recombinant goldfish antigen production and Western blot validation to conclusively confirm antibody specificity. While our study provides molecular and spatial evidence for distinct subsets of intestinal macrophages in goldfish, including the enrichment of *csf1rb* in intestine, and the localization of CSF/CSFR and BMP2⁺ macrophages in close association with enteric axons, we acknowledge that functional validation is required. Future studies will employ targeted assays such as CSF1/CSFR1 gain- and loss-of-function approaches in primary macrophage cultures and intestinal explants to assess survival and signaling pathways, and BMP2 modulation experiments in organ bath contractility assays to evaluate macrophage–neuron crosstalk. These experiments will directly test the mechanisms inferred from our current gene expression and immunohistochemical findings.

## Conclusions and perspectives

This study provides the first molecular and immunohistochemical characterization of intestinal macrophages expressing CSF, its receptor (CSFR), and bone morphogenetic protein 2 (BMP2) in the goldfish (*Carassius auratus*). Our findings reveal the presence of distinct macrophage populations localized in the mucosal, submucosal, and muscularis externa layers, suggesting specialized roles in gut immunity and neuromuscular regulation. The differential expression of *csf1a*, *csf1b*, *csf1ra*, and *csf1rb* further supports the functional heterogeneity of these cells. The presence of BMP2-expressing macrophages within the muscularis externa indicates a potential interaction with enteric neurons, drawing parallels with mammalian models of gut-immune crosstalk. These results contribute to the growing understanding of teleost intestinal immune regulation and establish a foundation for future investigations into the role of CSF, CSFR, and BMP2 in fish immune responses, gut homeostasis, and host-microbe interactions. While early studies in fish relied heavily on molecular tools (RT-PCR), recent research has employed mammalian antibodies to CSF1/CSF1R due to high conservation of the receptor’s intracellular tyrosine kinase domain across vertebrates, including fish. The main limitation is the lack of specific validated antibodies for all teleost fish species, forcing researchers to rely on mammalian counterparts that may not recognize all isoforms of the fish protein, especially the extracellular domain. Future investigations should prioritize species-specific antibody development and biochemical validation approaches to further strengthen immunocytochemical analyses in teleost systems.

## Supplementary Information

Below is the link to the electronic supplementary material.

Supplementary Information 1.

Supplementary Information 2.

Supplementary Information 3.

Supplementary Information 4.

## Data Availability

The datasets used and/or analyzed during the current study are available from the corresponding author upon reasonable request.
