# Supplementary figures and images for: Molecular and immunohistochemical characterization of intestinal macrophages subsets in goldfish
Source: Sci Rep. 2026 May 6;16:14397. doi: 10.1038/s41598-026-48801-y (PMC13149965; doi:10.1038/s41598-026-48801-y)

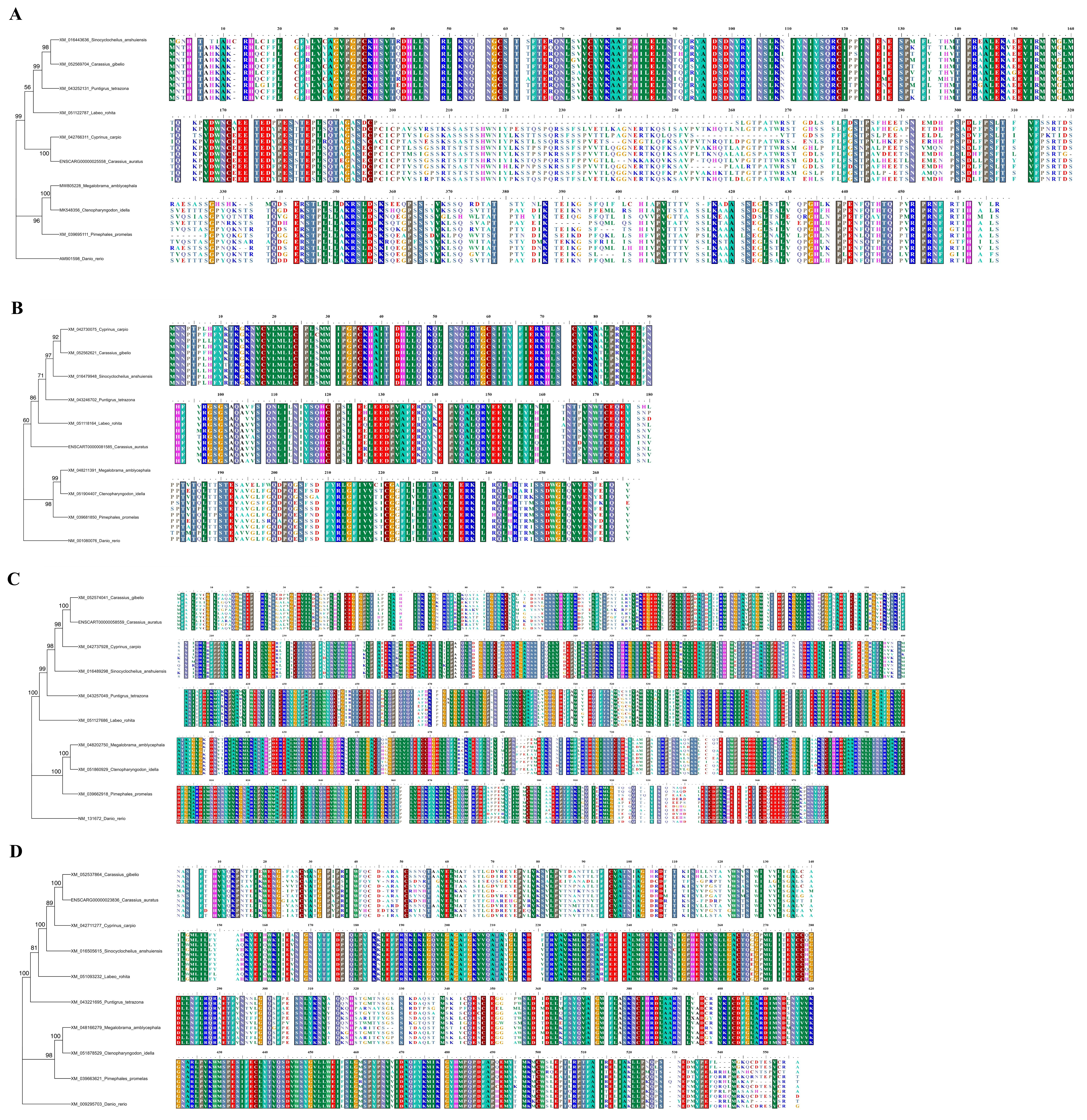

Supplement: Supplementary file 1 — Supplementary Information 1. [file 41598_2026_48801_MOESM1_ESM.jpg]

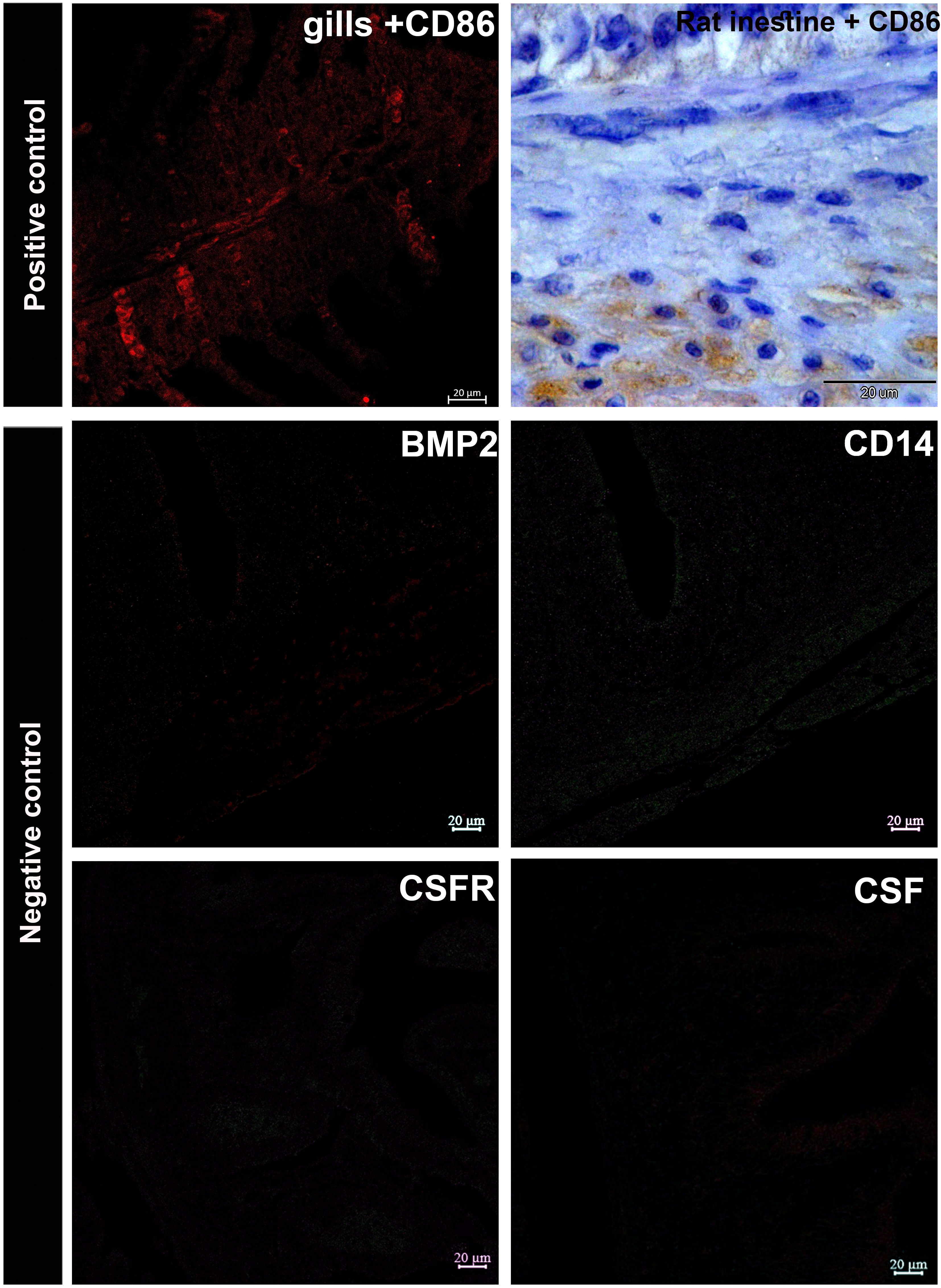

Supplement: Supplementary file 2 — Supplementary Information 2. [file 41598_2026_48801_MOESM2_ESM.jpg]
