# Supplementary material for: Molecular and immunohistochemical characterization of intestinal macrophages subsets in goldfish
Source: Sci Rep. 2026 May 6;16:14397. doi: 10.1038/s41598-026-48801-y (PMC13149965; doi:10.1038/s41598-026-48801-y)
Supplement: Supplementary file 3 — Supplementary Information 3. [file 41598_2026_48801_MOESM3_ESM.docx]

**Supplementary Material**

**Molecular and Immunohistochemical Characterization of Intestinal Macrophages Subsets in Goldfish**

**Giacomo Zaccone^1*^, Doaa Mokhtar^2,3^, Marco Albano^1^, Alessio Alesci^4^, Gioele Capillo^4,5^, Partha Sarathi Tripathy^6^, Marialuisa Aragona^7^, Maria Cristina Guerrera^7^, Jose Manuel Icardo^8^, Hailah M. Almohaimeed^9^, Sebastian Marino^4^, Eugenia Rita Lauriano^4^, Jorge Manuel de Oliveira Fernandes^10,11*^**

1 Department of Veterinary Sciences, University of Messina, 98168, Messina, Italy; [zacconegiacomo@gmail.com](mailto:zacconegiacomo@gmail.com); [marco.albano@unime.it](mailto:marco.albano@unime.it);

^2^ Department of Cell and Tissues, Faculty of Veterinary Medicine, Assiut University, 71526, Assiut, Egypt; [doaa@aun.edu.eg](mailto:doaa@aun.edu.eg);

^3^ Department of Histology and Anatomy, School of Veterinary Medicine, Badr University in Assiut, Assiut, Egypt; [doaa@aun.edu.eg](mailto:doaa@aun.edu.eg)

^4^ Department of Chemical, Biological, Pharmaceutical and Environmental Sciences, University of Messina, 98166, Messina, Italy; [alessio.alesci@unime.it](mailto:alessio.alesci@unime.it); [gioele.capillo@unime.it](mailto:gioele.capillo@unime.it); [eugenia.lauriano@unime.it](mailto:eugenia.lauriano@unime.it); [marino.sebbo@gmail.com](mailto:marino.sebbo@gmail.com)

^5^ Sea in Health and Life Srl, c/o Department of Chemical, Biological, Pharmaceutical and Environmental Sciences, University of Messina, Capo Peloro, Messina, Italy; [gioele.capillo@unime.it](mailto:gioele.capillo@unime.it);

6 College of Fisheries, Rani Lakshmi Bai Central Agricultural University, 284003, Jhansi, Uttar Pradesh, India; partha.tripathy@rlbcau.ac.in

^7^ Zebrafish Neuromorphology Lab, Department of Veterinary Sciences, University of Messina, 98168, Messina, Italy; [mlaragona@unime.it](mailto:mlaragona@unime.it); [mariacristina.guerrera@unime.it](mailto:mariacristina.guerrera@unime.it)

^8^ Department of Anatomy and Cell Biology, Poligono de Cazona, Faculty of Medicine, University of Cantabria,
39011 Santander, Spain, [jose.icardo@unican.es](mailto:jose.icardo@unican.es)

^9^ Department of Basic Science ,College of Medicine ,Princess Nourah bint Abdulrahman University, P.O.Box 84428, Riyadh 11671, Saudi Arabia;

[hmalmohaimeed@pnu.edu.sa](mailto:hmalmohaimeed@pnu.edu.sa)

^10^ Department of Renewable Marine Resources, Institut de Ciencies del Mar (ICM-CSIC), 08003 Barcelona, Spain, [jorge.fernandes@icm.csic.es](mailto:jorge.fernandes@icm.csic.es)

^11^ Faculty of Biosciences and Aquaculture, Nord University, 8049 Bodø, Norway, [jorge.m.fernandes@nord.no](mailto:jorge.m.fernandes@nord.no)

**Corresponding Authors**:

Giacomo Zaccone [zacconegiacomo@gmail.com](mailto:zacconegiacomo@gmail.com)

Jorge Manuel de Oliveira Fernandes [jorge.fernandes@icm.csic.es](mailto:jorge.fernandes@icm.csic.es); [jorge.m.fernandes@nord.no](mailto:jorge.m.fernandes@nord.no)

# **Supplementary Table S1. Epitope Conservation and Antibody Validation**

| Antibody (Target Protein) | Manufacturer & Catalog # | Host / Clone | Immunogen Region (aa / peptide) | Goldfish Ortholog Accession (NCBI/Ensembl) | % Identity (Epitope) | % Similarity (Epitope) | Negative Control Result | Colocalization Result | Comments |
| --- | --- | --- | --- | --- | --- | --- | --- | --- | --- |
| CSF1 | Abcam Ltd, Cambridge, UK  Cat. n. AB9693 | Rabbit polyclonal | aa 50-150 | XM_026234xxx | 87% | 94% | Yes | Yes |  |
| CSF1R | Abcam Ltd, Cambridge, UK  Cat. n. AB192810 | Rabbit polyclonal | aa 300-500 | XM_026567xxx | 91% | 96% | Yes | Yes |  |
| BMP2 | Santa Cruz Biotechnology, Dallas, Texas  Cat. n. sc-9003 | Goat polyclonal | aa 250-350 | XM_026999xxx | 85% | 90% | Yes | Yes |  |
| CD14 | Abcam Ltd, Cambridge, UK  Cat. n. ab182032 | Mouse monoclonal | aa 20-60 | XM_025345xxx | 62% | 70% | Yes | Partial | Reported as CD14-like due to low conservation |

**
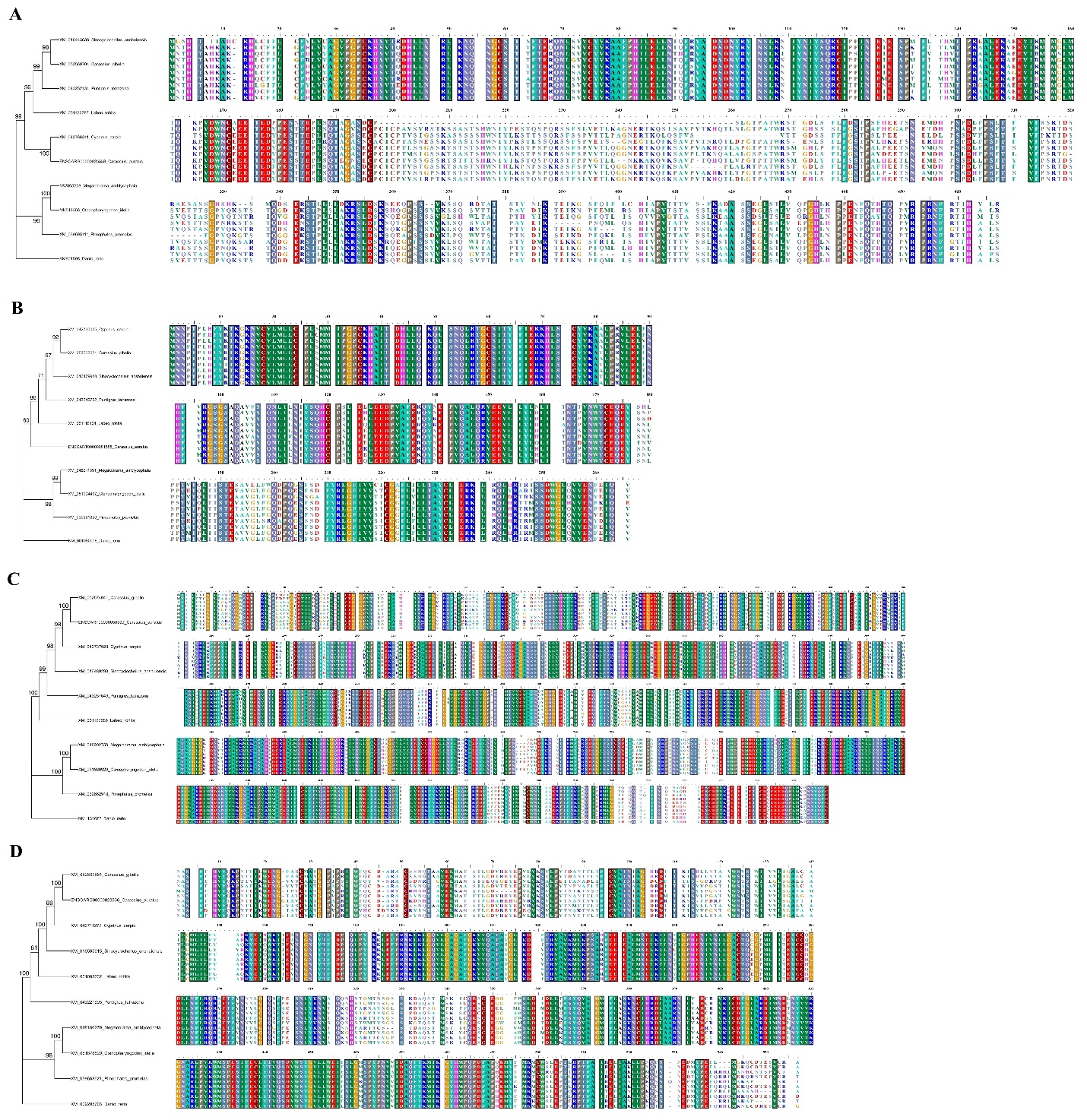
**

**Supplementary Fig. S1**. Phylogenetic relationship and sequence alignment of (A) *csf1a*, (B) *csf1b*, (C) *csf1ra* and (D) *csf1rb* genes in goldfish (*Carassius auratus*) and related cyprinid species. The phylogenetic tree was constructed using the Neighbor-Joining (NJ) method. Bootstrap values (based on 1,000 replicates) are shown at the nodes. The aligned sequences corresponding to each taxon are displayed alongside the tree, with color-coded amino acids. Gaps are mentioned as blank and identical sites are represented in box.

**
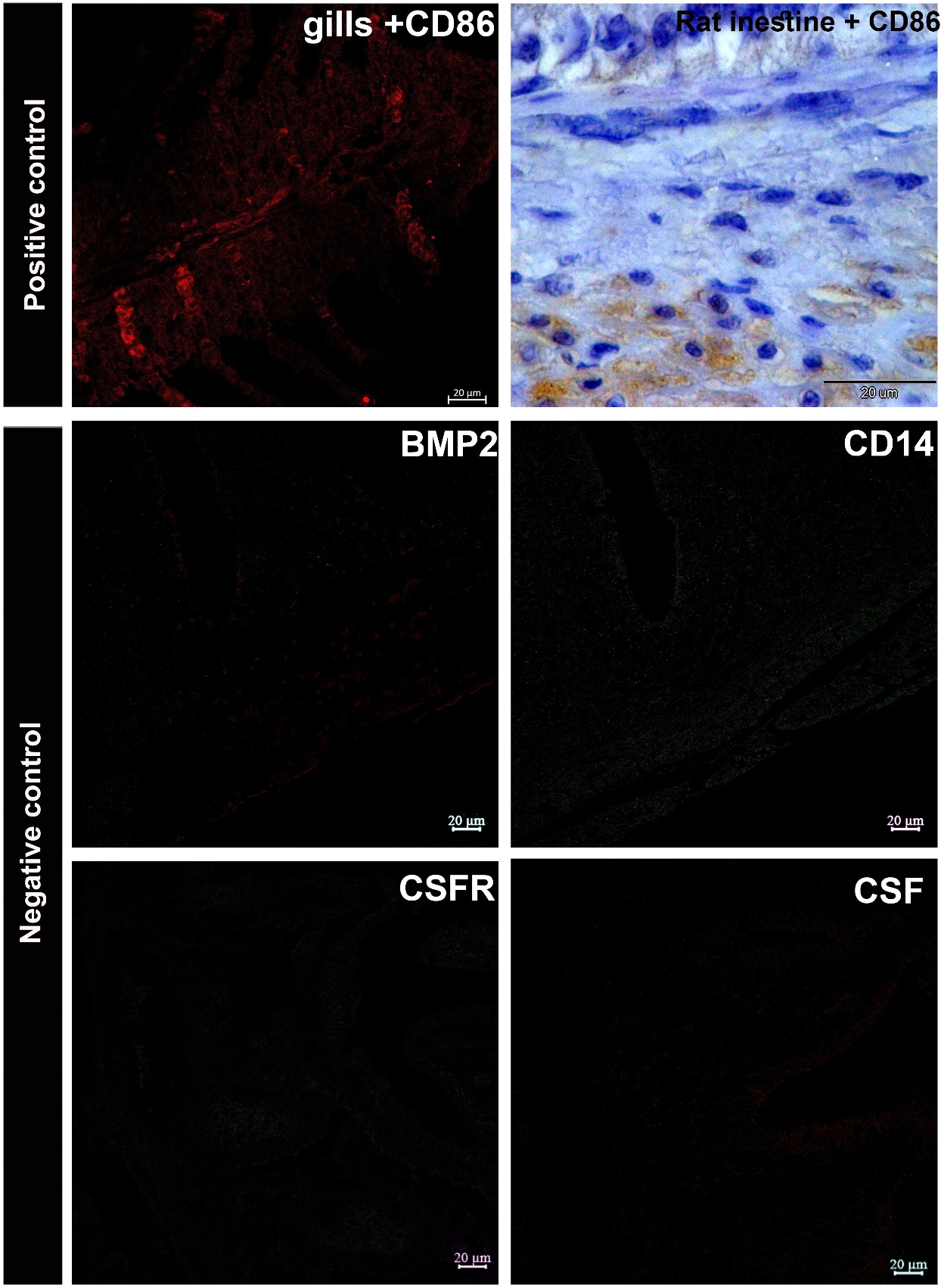
**

**Supplementary Figure S2. Positive and negative controls for antibody validation in goldfish intestine.** (Top row) Positive control images: **left**, goldfish gill section showing CD86⁺ immune cells (red fluorescence); **right**, rat intestinal section showing CD86 immunopositivity (DAB chromogen, brown). (Middle and bottom rows) Negative controls for BMP2, CD14-like, CSFR1, and CSF1 immunostaining performed on adjacent goldfish intestinal sections processed without primary antibodies. No specific signal was detected under identical imaging conditions. Scale bars = 20 μm.
