# Supplementary material for: Molecular and immunohistochemical characterization of intestinal macrophages subsets in goldfish
Source: Sci Rep. 2026 May 6;16:14397. doi: 10.1038/s41598-026-48801-y (PMC13149965; doi:10.1038/s41598-026-48801-y)
Supplement: Supplementary file 4 — Supplementary Information 4. [file 41598_2026_48801_MOESM4_ESM.docx]

# **Supplementary Table S1. Epitope Conservation and Antibody Validation**

| Antibody (Target Protein) | Manufacturer & Catalog # | Host / Clone | Immunogen Region (aa / peptide) | Goldfish Ortholog Accession (NCBI/Ensembl) | % Identity (Epitope) | % Similarity (Epitope) | Negative Control Result | Colocalization Result | Comments |
| --- | --- | --- | --- | --- | --- | --- | --- | --- | --- |
| CSF1 | Abcam Ltd, Cambridge, UK  Cat. n. AB9693 | Rabbit polyclonal | aa 50-150 | XM_026234xxx | 87% | 94% | Yes | Yes |  |
| CSF1R | Abcam Ltd, Cambridge, UK  Cat. n. AB192810 | Rabbit polyclonal | aa 300-500 | XM_026567xxx | 91% | 96% | Yes | Yes |  |
| BMP2 | Santa Cruz Biotechnology, Dallas, Texas  Cat. n. sc-9003 | Goat polyclonal | aa 250-350 | XM_026999xxx | 85% | 90% | Yes | Yes |  |
| CD14 | Abcam Ltd, Cambridge, UK  Cat. n. ab182032 | Mouse monoclonal | aa 20-60 | XM_025345xxx | 62% | 70% | Yes | Partial | Reported as CD14-like due to low conservation |
